# 

**Published:** 1985-01

**Authors:** 


Contents

1. Wordsworth, Presidential Address
Ian Bailey

10. Is there an Air Freshener Syndrome?
R. H. Lawson

14. Testicular Tumours, Treatment Study Group
Harold Eckert and Ann Light

18. Experiences in a Coagulation Referral Clinic in Bristol
Helen Daly and G. Wakerley

21. From our Correspondents

24. Surgical Club of South West England—Abstracts of Papers

29. Rectal Cancer—Report of Symposium

17. Book Review

